# Semi-Automated 3D Volumetric Renal Measurements in Polycystic Kidney Disease Using b0-Images—A Feasibility Study

**DOI:** 10.3390/tomography7040049

**Published:** 2021-10-10

**Authors:** Alexandra Roudenko, Soran Mahmood, Linda Du, Drew Gunio, Irina Barash, Florence Doo, Alon Slutzky, Nina Kukar, Barak Friedman, Alexander Kagen

**Affiliations:** 1 Department of Radiology, Allegheny Health Network, Pittsburgh, PA 15212, USA; 2 Department of Radiology, UT Health East Texas, Tyler, TX 75701, USA; soran.mahmood@uthct.edu; 3 Department of Radiology, Atrius Health, Boston, MA 02189, USA; linda.h.du@gmail.com; 4 Department of Radiology, New York Presbyterian, New York, NY 10021, USA; drew.gunio@gmail.com; 5 Department of Nephrology and Hypertension, Weill Cornell Medicine, New York, NY 10021, USA; irb9023@med.cornell.edu; 6 Department of Radiology, Mount Sinai West, New York, NY 10019, USA; Florence.Doo@mountsinai.org (F.D.); Alon.Slutzky@mountsinai.org (A.S.); Nina.Kukar@mountsinai.org (N.K.); Barak.Friedman@mountsinai.org (B.F.); alexander.kagen@mountsinai.org (A.K.)

**Keywords:** b0, kidney volume, polycystic kidney disease, MRI

## Abstract

Autosomal dominant polycystic kidney disease (ADPKD) eventually leads to end stage renal disease (ESRD) with an increase in size and number of cysts over time. Progression to ESRD has previously been shown to correlate with total kidney volume (TKV). An accurate and relatively simple method to perform measurement of TKV has been difficult to develop. We propose a semi-automated approach of calculating TKV inclusive of all cysts in ADPKD patients based on b0 images relatively quickly without requiring any calculations or additional MRI time. Our purpose is to evaluate the reliability and reproducibility of our method by raters of various training levels within the environment of an advanced 3D viewer. Thirty patients were retrospectively identified who had DWI performed as part of 1.5T MRI renal examination. Right and left TKVs were calculated by five radiologists of various training levels. Interrater reliability (IRR) was estimated by computing the intraclass correlation (ICC) for all raters. ICC values calculated for TKV measurements between the five raters were 0.989 (95% CI = (0.981, 0.994), *p* < 0.01) for the right and 0.961 (95% CI = (0.936, 0.979), *p* < 0.01) for the left. Our method shows excellent intraclass correlation between raters, allowing for excellent interrater reliability.

## 1. Introduction

Rising healthcare expenditures—both on a personal [1] and national level [2,3]—are no surprise to anyone. Chronic diseases, such as chronic kidney disease (CKD), account for a significant source of steady expenditures, with CKD affecting approximately 26 million people in the United States [4]. The costs associated with CKD only increase with increasing disease stage severity [5], as patients inevitably progress toward end stage renal disease (ESRD). In patients with multiple comorbidities such as diabetes and hypertension contributing to their CKD, treatment is usually aimed at the underlying conditions in the hopes of limiting their progression to CKD. However, hereditary disorders present a different challenge. Many hereditary disorders, for example Alport syndrome, steroid resistant nephrotic syndrome, and nail-patella syndrome, have been shown to be due to single gene mutations [6]. Ideally, the treatment would be tailored toward the underlying genetic abnormality responsible for the patient’s disease, however such treatment is not always readily available. Of hereditary disorders leading to ESRD, autosomal dominant polycystic kidney disease (ADPKD) is the most common [7] where patients slowly develop cysts that compromise their renal function. The progression to ESRD in ADPKD patients has been previously shown to be correlated with total kidney volume [7,8] (TKV) as the higher number and increasing size of cysts continuously compromises renal function [9,10].

An accurate and relatively simple method to perform measurement of kidney volume has been difficult to develop. Ultrasound methods are limited, especially in kidneys >17 cm in length [9], and the Consortium for Radiologic Imaging in Polycystic Kidney Disease (CRISP) cohort trial found MRI to be more accurate and reproducible compared to ultrasound [11]. Various MRI methods have been described for evaluating kidney volumes, including the use of coronal T1 weighted images with the use of a stereological method [10,12] as in the CRISP trial [10]. Alternative methods, including the use of T2 weighted images [13] and semi-automated segmentation of individual renal cysts, have been proposed, but those are limited in patients with high cyst burdens and more severe disease [14]. Additionally, relying solely on T2 weighted imaging methods can be problematic, as cysts are not uniformly T2 hyperintense, and those with hemorrhagic or proteinaceous components may appear dark on T2 weighted imaging. A study from 2013 by Bae et al. comparing manual, semi-automated, and region-based thresholding methods for calculating kidney volumes based on T2 weighted imaging noted that these T2 hypointense cysts would not be picked up by any of their employed methods [14]. However, these cysts would still restrict on diffusion weighted images and be included in our method of TKV calculation. Methods relying on stereological techniques, the use of the ellipsoid formula, or tracing the kidney freehand [15] can be time-consuming and cumbersome, increasing the examination interpretation time [16], which can be prohibitive if multiple examinations requiring TKVs are routinely performed.

Automated approaches are ideal as they require less time during a radiologist’s busy workflow, are more consistent, and preferably can be done at the PACS workstation. Prior studies utilizing cardiovascular MR examinations demonstrated that automated evaluation of various cardiac parameters including ventricular volume measurements are on par or superior to manual measurements performed by radiologists [17,18] and deliver important prognostic information and risk prediction, which is not significantly changed by manual correction [19]. A recently described automated approach on T1 weighted images has been compared to more cumbersome and time-consuming stereological and semi-automated approaches with great results [20]. However, given the variable signal characteristics of cysts on T1 weighted images as well many adjacent structures of similar signal and patients’ varied abilities to follow breathing instructions, this method may have its limitations, with a recent study of 40 patients demonstrating TKV evaluation on T2 images results in lower variability with images more often of sufficient quality [21]. We propose a semi-automated approach of TKV calculation based on b0 images, which allow for the greatest contrast between the hyperintense kidneys and cysts and the remainder of the visualized adjacent intraabdominal structures. This sequence is already routinely performed as part of our renal mass protocol MRI abdomen, and therefore no additional sequences or time would need to be added to the exam time. Additionally, this technique is relatively quick to perform and therefore is not expected to significantly extend a radiologist’s interpretation of the MRI examination. To the best of our knowledge, there have been no previously described semi-automated methods of TKV calculation using the b0 sequence. This software package has been used for other volumetric calculations of the liver and spleen with accurate and rapid results that were not significantly changed with time-consuming manual corrections [22] as well as with CT images to calculate the volume of renal masses and remaining renal parenchyma prior to nephrectomy [23].

Our purpose in this study is to evaluate the reliability and reproducibility of calculating TKVs, inclusive of all renal cysts, in ADPKD patients, using our semi-automated b0 method. This will be performed by raters of various training levels within the environment of an advanced 3D viewer.

## 2. Materials and Methods

### 2.1. Study Design and Recruitment

An institutional review board waiver was granted for this retrospective feasibility study at our tertiary care institution (approved 7/27/17 by Mount Sinai, HS#17-00353, GCO# 17-0854(00001)). Patients with polycystic kidney disease who have been referred by a nephrologist specializing in ADPKD for an MRI abdomen examination for evaluation of their kidneys were retrospectively recruited through a PACS database search. The period of recruitment was from November 2014 to October 2016. Secondary to the nature of the study, all men, women, minorities and their subpopulations were included who have been referred to the tertiary care site for an MRI examination of the kidneys with a diffusion weighted sequence being included as part of their evaluation. Patients who did not have diffusion weighted imaging as part of their MRI examination were excluded. Collected demographic data included patient age and sex.

### 2.2. MRI Examination

Multichannel MRI systems were used for scanning on 1.5-T MR imaging systems (GE HDx platform 15.0 or higher; GE Healthcare, Waukesha, WI, USA). Routine sequences performed included nonfat suppressed transverse and coronal single-shot fast spin echo T2WI (SSFSE), T1WI in- and out-of-phase, and DWI, and many patients had dynamic multiphase T1WI also performed. T2 weighted imaging utilizing SSFSE sequences with parameters: TR range: 1200–1500, TR median: 1500, TE range: 87–92, TE median: 91. Respiratory triggered diffusion weighted imaging was performed as an EPI-2D sequence with fat suppression performed with water excitation RF pulse; both b0 and b600 were acquired (TR range: 588–11,250 ms, TR median: 11,250 ms, TE range: 29–90 ms, TE median: 68 ms, Slice: 5–7 mm). However, only b0 images were used, as it allows the for the greatest contrast and signal-to-noise (SNR) ratio of cysts to background, thereby allowing the “organ tool” on the 3D advanced viewer to auto select all of the cysts.

### 2.3. TKV Calculation

**Figure 1:**
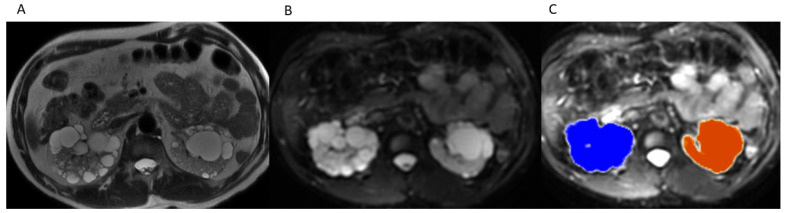
(**A**) Axial T2WI without fat suppression through kidneys and cysts. (**B**) Axial b0 through kidneys and cysts. (**C**) Outline of organ of interest overlaid on b0 in 3D Advanced Viewer.

**Figure 2:**
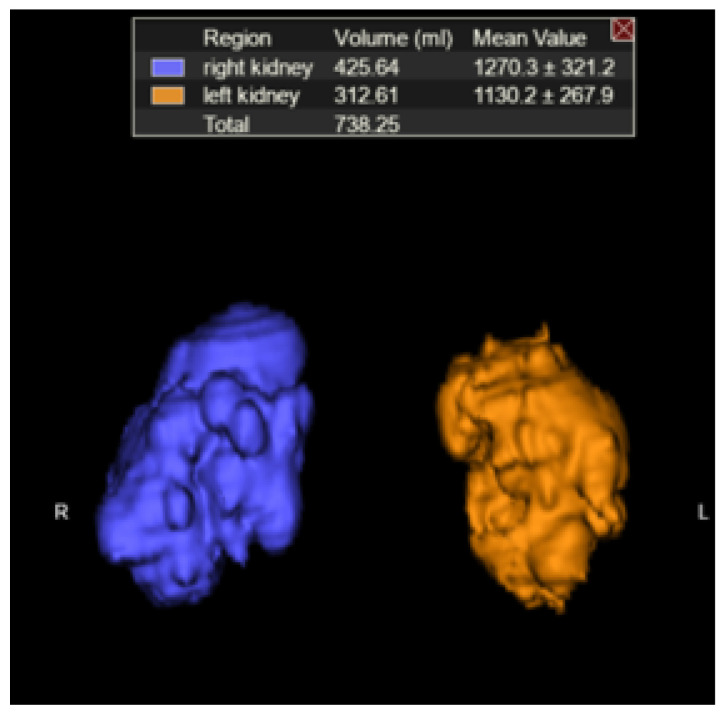
Kidneys plus cysts and estimated TKV (3D rendering).

Right and left TKVs were calculated by raters of varying training levels (attending with 10 years of experience, PGY-2 resident, PGY-3 resident, PGY-4 resident, PGY-5 resident) using Vitrea Software (Vital Imaging, Minnetonka, MN, USA). Each rater loaded the MRI abdomen examination into an advanced 3D viewer (Vitrea, Vital Imaging, Minnetonka, MN, USA). Using the b0 sequence and the “Segment Anatomy Tool” under the “Organ” setting, each rater highlighted the kidneys and cysts contained within them in a semi-automated fashion. The rater would then confirm the highlighted segments included the kidneys and cysts and had the option to exclude surrounding structures if inappropriately highlighted (Figure 1), which mostly came into play with the left kidney due to the adjacent spleen. The spleen is of similar intensity on the b0 images and would occasionally be erroneously highlighted. Utilizing the ‘undo’ button, the rater would remove the last addition and then redo the highlighting. If necessary, on rare occasions, the spleen can also be manually segmented out utilizing the software. The rater would then select “show volume” (under “options”) and the calculated volume would be displayed on the 3D panel (Figure 2).

### 2.4. Statistical Methodology

Interrater reliability (IRR) was estimated by calculating intraclass correlation (ICC) coefficients [24]. ICC was calculated for all raters on right and left TKV data sets separately using R Studio software [25] utilizing the irr software package [26] with model: twoway and type: agreement.

### 2.5. Accuracy Testing

MR images were obtained of saline-filled phantoms of known volumes (500 mL, 1 L), and the same method as delineated in Section 2.3 was performed as well as a manual freehand planimetry method to compare the calculated volumes.

## 3. Results

For the 30 subjects in our study, there were 19 females and 11 males. The average patient age was 49 (range (27, 65), median 49). The average right TKV for all patients was 832 mL (range (132, 2378)) and 698 mL (range (147, 3054)) for the left. Average time for calculating TKV’s was 3.2 min (range 1–9 min, median 3 min, *n* = 18).

Single score intraclass correlation was calculated for right and left TKVs using twoway model and agreement type with R Studio for the 30 subjects and 5 raters. ICC coefficients for right TKV’s was 0.989 (95% CI = (0.981, 0.994), *p* value < 0.01) and 0.961 (95% CI = (0.936, 0.979), *p* < 0.01) for left TKV’s (Table 1) consistent with statistically significant excellent interrater reliability [24].

Manual freehand and semi-automated calculations for phantom total volumes yielded 558 and 1097 mL, respectively, using the organ tool method described above and 618 and 1172 mL utilizing manual freehand planimetry. The organ tool method took one click and less than 5 seconds, while the manual freehand method required multiple clicks, drags and adjustments and took approximately 5 min.

## 4. Discussion

In our feasibility study, we presented a novel method of calculating TKVs in patients with ADPKD, which we believe offers several advantages compared to prior methods. Additionally, we were able to demonstrate excellent IRR as estimated with ICC as calculated using R Studio.

**Figure 3:**
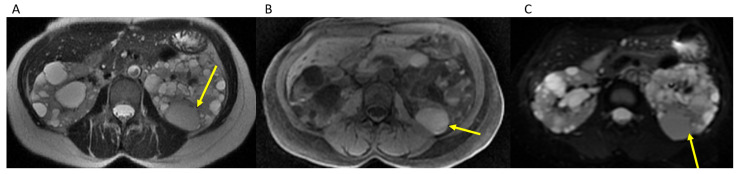
Hemorrhagic cyst (yellow arrow) remains brighter than background on b0 images (**A**). Axial T2WI through kidneys and cysts. (**B**). Axial T1 without contrast through kidneys and cysts. (**C**). Axial b0 through kidneys and cysts.

Our method addresses limitations of previously described methods relying solely on dedicated T2 weighted sequences, as cysts with hemorrhagic or proteinaceous components would be T2 hypointense and would not likely be included in the calculation method employed to estimate kidney volume, as noted by Bae et al. [14], but would still remain bright on b0 images and be included in the TKV measurement utilizing the semi-automated organ tool method (Figure 3). These more complex cysts are also more common in patients with more severe disease. Although our method cannot differentiate between renal tissue and cysts, studies have shown that it is the overall kidney size and volume inclusive of cysts which is correlated with progression to ESRD [7,10,27]. Additionally, our method is not limited to patients with mild to moderate ADPKD similar to previously described segmentation methods [14] and can be used for kidneys of any size as long as the entire kidney is included on the b0 images, which they should be for renal MRI examination protocols. Additional advantages of our method include no additional sequences to be performed or time required on the MRI scanner outside of our usual protocol for renal MRI examinations. Sharma et al. demonstrated the highest accuracy with planimetry on MR images, although they used T2, FIESTA, ad FISP images which, while providing great detail, also makes it harder, as the signal intensity between the kidney and cysts is similar to adjacent organs. Their fastest planimetry method was a freehand method, which required 20 min for MR [15]. Although less accurate for MR examinations as per the results of Sharma et al., their fastest method required 11 min for stereological techniques [15]. By utilizing the high contrast between the kidney and cysts and the background structures on the b0 images, we can drastically reduce the time to average of 3.2 min (rage 1–9 min) for each case and there are no calculations to perform for the interpreting radiologist, unlike prior methods using the ellipsoid formula or stereological methods [15]. Moreover, our method is relatively intuitive with a simple click-on-the-organ-of-interest method requiring minimal additional exam interpretation time. The required 3D advanced viewer software we currently have installed on all of our workstations is routinely used for other applications within our department, although we recognize that it is a proprietary third-party software, instead of freeware that is more widely available but would require the use of a potentially more time-consuming manual segmentation method. Lastly, given the excellent IRR amongst raters of various training levels including junior residents, this is accessible to residents to perform while on their rotations, further assisting in the time constraints on an attending radiologist.

We believe our method can be easily integrated into routine clinical practice in the radiologic management of patients with ADPKD given its accessibility and ease of use. The ability to accurately measure TKVs and monitor disease progression will become more crucial as new treatments are developed for patients with ADPKD. As prior studies have shown [7,28], total kidney volume has been shown to be correlated with progression to ESRD; however, there is significant inter-individual variability in the rate of TKV growth [16]. An ability to easily track TKV without considerably extending MRI examination interpretation time will become more crucial to monitor treatment response and disease progression as new treatments enter the market.

Our limitations include a small sample size of 30 subjects; however, Koo et al. suggests a minimum of 30 subjects and three raters as a good rule of thumb for reliability studies [24]. Additionally, we did not compare our method with prior methods reported in the literature, for example, stereological methods or using the ellipsoid formula; however, we performed the same technique on phantoms of known volumes and compared the results to those obtained using a manual freehand method.

## 5. Conclusions

Our method offers several advantages in calculating TKVs, including demonstrating excellent reliability while requiring minimal extra time on the part of the reading radiologist. This allows radiologists to provide the treating clinician with an additional tool in managing their patients with ADPKD, especially as new treatments are developed and come to market.

## Figures and Tables

**Table 1:** Statistical analysis results.

	RIGHT	LEFT
ICC	0.989	0.961
95% CI	[0.981, 0.994]	[0.936, 0.979]
*P* VALUE	<0.01	<0.01

## Data Availability

The data presented in this study are available on request from the corresponding author. The data are not publicly available due to Health Insurance Portability and Accountability Act (HIPAA) policies and restrictions.
